# Nonoxidative ethanol metabolism in humans—from biomarkers to bioactive lipids

**DOI:** 10.1002/iub.1569

**Published:** 2016-10-06

**Authors:** Christoph Heier, Hao Xie, Robert Zimmermann

**Affiliations:** ^1^ Institute of Molecular Biosciences, University of GrazAustria

**Keywords:** nonoxidative ethanol metabolism, ethyl glucuronide, ethyl sulfate, phosphatidylethanol, fatty acid ethyl ester

## Abstract

Ethanol is a widely used psychoactive drug whose chronic abuse is associated with organ dysfunction and disease. Although the prevalent metabolic fate of ethanol in the human body is oxidation a smaller fraction undergoes nonoxidative metabolism yielding ethyl glucuronide, ethyl sulfate, phosphatidylethanol and fatty acid ethyl esters. Nonoxidative ethanol metabolites persist in tissues and body fluids for much longer than ethanol itself and represent biomarkers for the assessment of ethanol intake in clinical and forensic settings. Of note, the nonoxidative reaction of ethanol with phospholipids and fatty acids yields bioactive compounds that affect cellular signaling pathways and organelle function and may contribute to ethanol toxicity. Thus, despite low quantitative contributions of nonoxidative pathways to overall ethanol metabolism the resultant ethanol metabolites have important biological implications. In this review we summarize the current knowledge about the enzymatic formation of nonoxidative ethanol metabolites in humans and discuss the implications of nonoxidative ethanol metabolites as biomarkers of ethanol intake and mediators of ethanol toxicity. © 2016 IUBMB Life, 68(12):916–923, 2016

## Introduction

The consumption of ethanol has a widespread social tradition among many populations worldwide. Whereas moderate ethanol intake has been regarded beneficial to cardiovascular health, chronic alcohol abuse is associated with an increased risk of pancreatitis, cardiomyopathy, liver disease and cancer 1. Although the cellular and molecular etiology underlying ethanol‐associated diseases is incompletely understood a causative role has been attributed to the metabolic conversion of ethanol resulting in the generation of toxic intermediates and metabolic stress. After absorption by the oral, gastric and intestinal mucosas and distribution via the circulation the majority of ingested ethanol (95–98%) is metabolized and only a small fraction is excreted unchanged via breath, urine and sweat. The prevalent route of human ethanol metabolism is hepatic ethanol oxidation, which eliminates more than 90% of the ingested ethanol. The first step in the oxidative pathway is the conversion of ethanol to acetaldehyde catalyzed by cytosolic alcohol dehydrogenase and to a lesser extent by microsomal cytochrome P450 isoforms (especially CYP2E1) and catalase. In a second step, acetaldehyde dehydrogenase oxidizes acetaldehyde to acetate, which is mainly secreted into the circulation and converted to acetyl‐coenzyme A (CoA) by extrahepatic tissues such as muscle, heart and brain 2. In addition to oxidation, several nonoxidative routes of ethanol metabolism have been described that result in the enzymatic conjugation of ethanol to endogenous metabolites such as glucuronic acid, sulfate, phospholipids and fatty acids (FAs). The resultant metabolites are termed ethyl glucuronide (EtG), ethyl sulfate (EtS), phosphatidylethanol (PEth) and fatty acid ethyl ester (FAEE). In quantitative terms nonoxidative pathways constitute a minor fraction of total ethanol metabolism 2. However, due to slower elimination rates nonoxidative ethanol metabolites persist in body fluids and tissues for much longer than ethanol itself. This characteristic makes nonoxidative ethanol metabolites biomarkers that enable the retrospective assessment of ethanol intake even when ethanol itself is no longer present in the body. Moreover, accumulating evidence suggests that formation of specific nonoxidative ethanol metabolites interferes with cellular signaling pathways, disrupts organelle function and contributes to ethanol toxicity in organs with limited oxidative capacity. In the following sections we will review the biochemistry and biological significance of nonoxidative ethanol metabolism and discuss the implications of nonoxidative ethanol metabolites as biomarkers for ethanol intake and mediators of ethanol toxicity.

## Ethyl Glucuronide and Ethyl Sulfate

### Formation and Tissue Distribution

**Figure 1:**
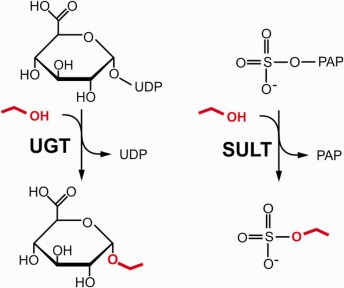
Enzymatic formation of EtG and EtS. EtG is formed by transfer of a glucuronyl moiety from UDP‐glucuronic acid to ethanol (marked red) catalyzed by UGT. EtS is formed by transfer of a sulfonate group from 3′‐phosphoadenosine‐5′‐phospho (PAP) sulfate to ethanol catalyzed by SULT.

EtG is formed by transfer of a glucuronyl moiety from uridine 5′‐diphospho (UDP)‐glucuronic acid to ethanol (Fig. 1). This reaction is catalyzed by UDP‐glucuronosyltransferases (UGTs), an enzyme family involved in phase II metabolism of xenobiotics as well as glucuronidation of endogenous metabolites 3. EtG was first identified in urine of ethanol‐intoxicated rats and afterwards in blood and urine of ethanol‐consuming humans 4, 5. Blood EtG can be detected 1 h after beginning of ethanol intake, and peak concentrations are typically reached between 3.5 and 5.5 h 6, 7. In addition to blood, detectable EtG levels have been reported in several other tissues including adipose tissue, liver, brain, bone marrow, muscle and hair 8, 9. Excretion of EtG occurs mainly via the urine and was shown to account for a minor part (<0.1%) of total ethanol elimination 10. EtS is formed by sulfonation of ethanol catalyzed by another class of phase II enzymes termed sulfotransferases (SULTs, Fig. 1). Like EtG, EtS was first observed in rats after ethanol administration and later detected in urine of ethanol‐consuming human subjects 11, 12. The formation, pharmacokinetics and elimination of EtS are similar to EtG and <0.1% of the ingested ethanol is typically excreted as urinary EtS 10. In addition to blood and urine, EtS has also been detected in liver, kidney, placenta, fetal tissues and hair 13, 14, 15.

### Enzymes Catalyzing EtG and EtS Formation

The human genome encodes for 22 UGTs, which are divided into three subfamilies, termed UGT1A, UGT2A and UGT2B. UGTs are localized at the endoplasmic reticulum with active sites facing the lumen. Each UGT isoform shows a tissue‐specific expression pattern. Liver, gastrointestinal tract and kidney express the highest levels of UGT isoforms 3. Consistent with the tissue expression and subcellular localization of UGTs, high ethanol glucuronidation activities have been measured in microsomal preparations of human liver and kidney 16. In addition, several UGT1A and UGT2B family members have been shown to catalyze EtG formation *in vitro*. Among them, UGT2B7 and UGT1A9 exhibited highest ethanol glucuronidation activities 16, 17. Based on inhibitor experiments, these isoforms were estimated to account for ∼50% of ethanol glucuronidation activity in human liver microsomes 16, 18. Notably, the reported *K*_m_ values of ethanol glucuronidation exceed physiologically attainable ethanol concentrations 16, 19. These observations likely reflect low affinity of UGTs for ethanol and may explain the limited contribution of glucuronidation to overall ethanol metabolism. The enzymatic sulfonation of ethanol was first observed in rat liver samples and was later also measured in tissue extracts of human liver, intestine and lung 11, 20. To date 14 human SULT isoforms with different substrate specificities and tissue expression patterns have been described. SULTs catalyze the sulfonation of xenobiotics and endogenous metabolites using 3′‐phosphoadenosine‐5′‐phosphosulfate as donor 21. According to *in vitro* studies using recombinant SULTs, members of the 1A, 1B, 1C, 1E and 2A subfamilies are able to catalyze sulfonation of ethanol. Based on its high expression in liver SULT1A1 has been suggested to be a major contributor to hepatic EtS formation 22. However, the relative contribution of SULT isoforms to EtS formation *in vivo* is currently unknown.

### EtG and EtS as Biomarkers of Ethanol Intake

EtG and EtS exhibit extended half‐lives in body fluids as compared to nonmetabolized ethanol and have been used as biomarkers for recent ethanol intake and abstinence monitoring. After a single event of ethanol intake the time frame of detectable serum EtG and EtS exceeds that of blood ethanol by 4–8 h 6, 7. Moreover, urinary EtG and EtS remain detectable for 22–48 h after a single ethanol intake and for 40–130 h in heavy drinkers after withdrawal. As a consequence, determination of EtG and EtS in serum or urine permits verification of ethanol consumption even after ethanol is no longer detectable 6, 7, 23, 24. Further matrices for the detection of EtG (and EtS) are sweat, oral fluid, hair, meconium and placenta 14, 25, 26, 27, 28. The deposition of EtG in hair occurs through blood or sweat and is stable for several months thus permitting retrospective assessment of ethanol intake even after long periods of abstinence 29. Correlations between the amount of ingested ethanol and hair EtG concentrations have been observed in rodent models and human subjects, and threshold levels have been proposed to discriminate chronic excessive ethanol consumption from moderate drinking and abstinence 29, 30, 31.

### Biological Significance of EtG and EtS

Phase II modifications increase water solubility and facilitate excretion of metabolites 3. Hence, EtG and EtS are not considered as bioactive metabolites. EtG as well as glucuronic acid have recently been demonstrated to activate toll like receptor (TLR) 4 signaling *in vitro* and to cause allodynia in rats after intrathecal administration 32. However, whether EtG concentrations obtained after ethanol consumption are sufficient to activate TLR4 signaling *in vivo* remains to be established.

## Phosphatidylethanol

### Formation and Tissue Distribution

PEth is formed by transphosphatidylation of phospholipids with ethanol, which was first observed by Alling et al. 33 in ethanol‐intoxicated rats. PEth is detectable 2 h after acute ethanol intoxication in a multitude of rat tissues with highest levels in gastrointestinal tract, liver, lung and kidney 34. A similar tissue distribution of PEth deposition was also demonstrated in post‐mortem tissue samples of human subjects intoxicated with ethanol 34, 35. Furthermore, PEth can be found in blood of social drinkers and alcoholics and can thus serve as biomarker of ethanol intake (see below). After a single ethanol dose, blood PEth levels increase immediately reaching peak concentrations approximately 90–120 min after termination of drinking. With decreasing blood ethanol concentrations PEth levels decline with an estimated half‐life of 3–5 days but remain detectable for up to 28 days after sobriety 36, 37. The half‐life of PEth is tissue‐specific ranging from 1–2 h in pancreatic islets and perfused hearts to 10–17 h in brain 38, 39, 40.

### Enzymatic Formation and Degradation of PEth

**Figure 2:**
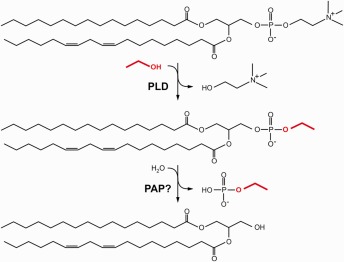
Enzymatic formation and degradation of PEth. PLD catalyzes transphosphatidylation of phosphatidylcholine and ethanol (marked red) resulting in the formation of PEth and the release of choline. PEth may be further metabolized by phosphatidate phosphohydrolase (PAP) to diacylglycerol and ethyl phosphate.

The transphosphatidylation of phospholipids and ethanol is catalyzed by phospholipase D (PLD) 41, 42. Under physiological conditions, PLD catalyzes hydrolysis of membrane phospholipids (predominantly phosphatidylcholine, PC) to phosphatidic acid (PA). PLD‐generated PA serves as lipid messenger and has been implicated in several important cellular processes such as membrane trafficking, cytoskeletal reorganization, endocytosis, proliferation and migration 43. In the presence of ethanol and other primary alcohols PLD performs a transphosphatidylation reaction resulting in the formation of PEth or other phosphatidylalcohols (Fig. 2). Interestingly, transphosphatidylation is more efficient than hydrolysis and has therefore been routinely used to measure PLD activity *in vitro* 43. In cultured cells, the rate of PEth formation correlates with ethanol concentrations in the medium and is promoted by pharmacological activation of PLD 44, 45, 46. Several studies indicate that PEth is mainly formed from PC 47, 48. Both, PLD1 and PLD2, two major mammalian PLD isoforms, have been shown to catalyze PEth formation *in vitro* 49. However, the contribution of each isoform to PEth synthesis *in vivo* has not been addressed. Cell culture studies indicate that PEth turnover occurs at slower rates compared to PA indicating that PEth is more resistant to further metabolic conversions 38, 50. Several phospholipases have been implicated in PEth breakdown including phospholipase A_2_, PC phospholipase C and PA phosphohydrolase 38, 51, 52. Propanolol, an inhibitor of PA phosphohydrolase, has been shown to block PEth turnover in rat pancreatic islets and human hepatoma cells indicating a major role of this enzyme in PEth degradation (Fig. 2) 38, 52. This notion is further supported by the presence of ethyl phosphate, a possible product of PA phosphohydrolase‐mediated PEth hydrolysis, in ethanol‐intoxicated rats 53.

### PEth as Biomarker of Ethanol Intake

Due to slow PEth elimination rates, detection of blood PEth permits verification of ethanol intake even after several days of abstinence. The majority of blood PEth is associated with erythrocytes whereas only a minor fraction can be found in leukocytes and plasma 54. As an alternative to whole blood samples PEth was also successfully detected in dried blood spots 55. Blood PEth levels were found to correlate with reported ethanol intake of alcoholic subjects and threshold levels considered typical of chronic ethanol abuse have been proposed to discriminate between moderate drinking and chronic ethanol abuse 10. Other matrices suitable for the detection of PEth are exhaled breath and umbilical cord 55, 56.

### Biological Significance of PEth

Because PEth formation occurs at the expense of PA upon activation of PLD it has been suggested that this reaction interferes with PLD‐mediated cellular processes 44, 45. In line with this notion, PEth formation was shown to inhibit PLD‐dependent mitogenic effects of serum and carbachol in astrocytes 46, 57. Conversely, PEth accumulation was shown to promote intestinal hyperplasia suggesting that cellular PEth formation can have promitotic and antimitotic effects 58. Moreover, PEth formation has been associated with increased basal levels of inositol 1,4,5‐trisphosphate, which is derived from the action of PLC indicating that PEth may influence other phospholipid signaling pathways apart from the PLD/PA axis 59. In line with this notion, several studies indicate that PEth directly affects structural properties of biomembranes and alters the function of membrane‐associated proteins. For example, inclusion of PEth in artificial or natural phospholipid membranes increases membrane fluidity and alters vesicle fusion in response to Ca^2+^ 60, 61. Furthermore, PEth was shown to affect the activity of several transporters and enzymes *in vitro* including Na^+^/K^+^‐ATPase, protein kinase C, phospholipase C and cytosolic phospholipase A_2_ 60, 62, 63, 64, 65. Taken together, these findings suggest that PEth formation affects cellular signaling pathways by multiple mechanisms including competition with PA synthesis and functional disturbance of biomembranes and membrane‐associated proteins.

## Fatty Acid Ethyl Ester

### Formation and Tissue Distribution

FAEEs are formed through the enzymatic esterification of ethanol with FAs. These ethanol metabolites have been described first by Goodman and Deykin in total body lipid extracts of rats acutely intoxicated with ethanol and were later found in multiple tissues of rodents subjected to acute or chronic ethanol exposure 66, 67, 68. In the 1980s, the presence of FAEEs was first detected in post‐mortem tissue samples of human subjects acutely intoxicated with ethanol 69 and up to now, a range of different tissues has been shown to possess FAEE synthetic activity *in vitro* and to accumulate FAEE *in vivo* after ethanol intake. Among them, highest FAEE levels have been consistently reported in liver and pancreas. Detectable amounts of FAEE are formed within minutes in cultured cells and perfused organs and dose–response relationships have been observed for FAEE synthesis rates and extracellular ethanol concentrations 70, 71, 72. Consistent with this, oral intake of ethanol rapidly increases blood FAEE levels in humans within several minutes, and peak concentrations are usually reached within a few hours closely paralleling blood ethanol levels 73. Rapid onset of FAEE formation and deposition was demonstrated also in other tissues such as placenta, heart, liver and brain 74, 75, 76. With the elimination of ethanol from the body, FAEE levels in blood and other tissues decline 73, 76. Yet, detectable amounts of FAEE persist in human blood for 24 h (social drinkers) to 99 h (heavy drinkers) and in rodent tissues for several days after termination of ethanol intake 73, 74, 75, 77.

### Enzymatic Formation and Degradation of FAEE

**Figure 3:**
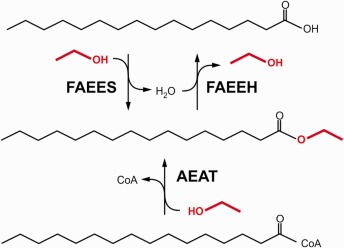
Enzymatic formation and degradation of FAEE. FAEE is formed by esterification of FA with ethanol (marked red) catalyzed by FAEES or by transfer of an acyl moiety from acyl‐CoA to ethanol catalyzed by AEAT. FAEEH catalyzes hydrolysis of FAEE to FA and ethanol.

Two enzyme classes catalyzing FAEE synthesis have been identified: FAEE synthase (FAEES) esterifies ethanol with free FAs whereas acyl‐CoA‐ethanol‐*O*‐acyltransferase (AEAT) transfers acyl moieties from acyl‐CoA to ethanol (Fig. 3) 69, 78, 79. In addition, FAEE may also form upon “ethanolysis” (instead of hydrolysis) of ester bonds in glycerolipids such as triacylglycerol or phospholipids catalyzed by (phospho)lipases 80, 81. FAEES activity has been found in soluble and microsomal fractions of multiple tissues with highest activities in pancreas and liver 69, 79, 82, 83. Inhibitor studies suggest that FAEES enzymes belong to the serine hydrolase family 83, 84, 85. Accordingly, several serine hydrolases with FAEES activity have been identified including carboxylesterases from heart, liver and adipose tissue, cholesterol ester hydrolase and triacylglycerol lipase from pancreas and lipoprotein lipase 81, 86, 87, 88, 89, 90, 91. Estimates on the contribution of these enzymes to tissue FAEES activities mainly rely on tissue expression patterns and enzyme inhibition studies. Tsujita and Okuda 80 removed FAEES activity from extracts of adipose tissue, testis, liver, small intestine and lung by immunoprecipitation with an antiserum generated against purified adipose tissue carboxylesterase indicating a possible function of this enzyme as FAEES. Interestingly, substantial residual FAEES activity was observed in liver, small intestine and kidney suggesting tissue‐specific contributions of different FAEES isoforms. The differential susceptibility of hepatic and pancreatic FAEES to organophosphates further supports this notion 92. However, as none of these *in vitro* studies has been verified in mutant animal models, the contribution of each FAEES enzyme to FAEE formation *in vivo* remains unknown. AEAT activity has been measured in homogenates and microsomal fractions of multiple tissues with highest levels in liver and small intestine 83, 93. Notably, AEAT activity was membrane‐associated and oriented mainly toward the lumen of isolated microsomes 68, 79, 93. Differential inhibition of AEAT and FAEES by several reagents indicates that these activities are mediated by distinct enzymes 83, 84. The cysteine‐reactive compound *p*‐chloromercuribenzenesulfonate inhibited both AEAT and acyl‐CoA hydrolase activities suggesting a relationship between both enzyme classes 79. However, unlike FAEES AEAT enzymes have not been purified from tissues and the molecular identity of AEAT remains elusive. FAEE hydrolase (FAEEH) catalyzes the cleavage of the ester bond within the FAEE molecule resulting in the release of FA and ethanol. This reaction may counteract FAEE formation or remove FAEE from cellular deposits and therefore likely determines the tissue half‐life of FAEE (Fig. 3). FAEEH activities have been measured in a multitude of tissues, and highest activities have been observed in pancreas and liver 83. Several serine hydrolases with FAEEH activity have been identified including hepatic carboxylesterase isoforms, hormone‐sensitive lipase and monoacylglycerol lipase (MGL) 84, 94, 95. Based on the abundance of carboxylesterase isoforms Diczfalusy et al. concluded that Es‐4 (also known as Ces1f) may be responsible for a considerable portion of rat hepatic FAEEH activity. However, the same group failed to detect expression of this isoform in human liver suggesting species‐specific differences in the molecular identity of hepatic FAEEH 83, 84. Recently, overexpression of MGL, a rate‐limiting enzyme for monoacylglycerol hydrolysis in multiple tissues, has been shown to attenuate FAEE accumulation in cultured cells incubated with ethanol. Conversely, pharmacological inhibition of MGL augmented intracellular FAEE suggesting that FAEE hydrolysis by MGL is a major determinant of cellular FAEE levels. Interestingly, MGL as well as FAEE were found to associate with lipid droplets (LDs), which are cellular storage organelles of hydrophobic esters such as triacylglycerol or steryl esters. This suggests that FAEE are transiently stored in LDs and hydrolyzed by LD‐associated MGL 95.

### FAEE as Biomarker of Ethanol Intake

FAEE depositions in hair have been proposed as suitable markers for the retrospective detection of alcohol abuse and the monitoring of abstinence 96. Hair FAEEs are most likely incorporated from sebum, which is produced by the sebaceous glands and regularly fats the hair shaft 97. FAEE can persist for month in hair of alcoholics even after a time of abstinence 97. Lower levels of FAEE are also found in hair of social drinkers, and traces are occasionally also detected in teetotalers likely reflecting FAEE formation from ectopically applied ethanol‐containing cosmetics 97. Typical for hair analysis, there is no clear relationship between consumed ethanol and hair FAEE levels. Besides differences in hair growth and ethanol metabolism also hair care and the use of cosmetics may affect hair FAEE concentrations. Nevertheless, suitable cutoff values have been established to discriminate heavy alcohol consumers from social drinkers 10, 97. FAEE have also been detected in meconium of newborns thus permitting determination of prenatal ethanol exposure 98. It has been shown that FAEE are unable to cross the placenta and hence derive from ethanol metabolism in the fetus 99. 75% of the FAEE detected in meconium reflect ethanol intake during the last 8 weeks of pregnancy suggesting that fetal ethanol exposure during early pregnancy may not be adequately detected by this approach 55. Other matrices for the detection of FAEE are blood and sebum 55, 77.

### Biological Significance of FAEE

Since the identification of FAEE in organs commonly damaged by ethanol such as heart, brain, pancreas and liver, an increasing number of studies linked FAEE formation to ethanol toxicity. Toxic cellular effects ascribed to FAEE include inhibition of cell proliferation, destabilization of lysosomes, mitochondrial depolarization and induction of apoptosis 100, 101, 102, 103, 104. Of note, infusion of FAEE or a combination of ethanol and FA into rodents elicited pancreatic edema, inflammation and necrosis resembling the characteristics of alcoholic pancreatitis 103, 105. Incubation of isolated pancreatic acinar cells with FAEE induced mitochondrial depolarization, depletion of cellular ATP and sustained elevations of intracellular Ca^2+^ levels ultimately associated with cellular dysfunction and cell death 106. Similar effects were observed when endogenous FAEE formation was promoted by incubating acinar cells with FA and ethanol in the presence of pharmacological inhibitors of ethanol oxidation. Of note, pharmacological inhibition of FAEE formation ameliorated pancreatic toxicity induced by FA and ethanol both *in vitro* and *in vivo* 103. At present it is unclear if FAEE itself or FAEE metabolites such as FA mediate FAEE‐associated cellular toxicity. It has been speculated that FAEE hydrolysis and the subsequent liberation of FA may be a critical molecular event through which FAEE induces cellular dysfunction 107, 108. This hypothesis is supported by a recent study showing that infusion of free FAs causes more severe pancreatic damage than infusion of the corresponding FAEE species 109. In addition to a possible role in mediating ethanol toxicity FAEEs have also been characterized as odor active substances that contribute to the flavor of food and beverages. Moreover, specific FAEE species act as pheromones in insects indicating that FAEEs can activate olfactory/pheromone receptors 110. It remains to be investigated whether FAEEs produced in response to alcohol consumption also act as receptor agonist contributing to psychoactive (or other) effects of ethanol.

## Conclusions

Although the quantitative contribution of nonoxidative pathways to human ethanol metabolism is low, the resultant metabolites have important analytical and biological implications. The detection of nonoxidative ethanol metabolites in body fluids, hair and neonatal matrices provides a valuable tool for the monitoring and retrospective assessment of ethanol intake. Different elimination rates of nonoxidative ethanol metabolites permit a wide range of analytical time frames for the verification of ethanol intake ranging from hours to months after termination of ethanol consumption. The enzymatic reaction of ethanol with cellular lipids generates bioactive metabolites such as FAEE and PEth, which have been shown to interfere with cellular signaling pathways and organelle function and may therefore contribute to specific manifestations of ethanol toxicity. Although considerable research has been performed regarding the enzymology of nonoxidative ethanol metabolism our current knowledge is limited mainly to *in vitro* studies. The characterization of enzymes and signaling pathways mediating effects of nonoxidative ethanol metabolites *in vivo* is thus inevitable to improve our understanding of nonoxidative ethanol metabolites as biomarkers and bioactive molecules.
